# Wound-Healing Potential of Cultured Epidermal Sheets Is Unaltered after Lyophilization: A Preclinical Study in Comparison to Cryopreserved CES

**DOI:** 10.1155/2013/907209

**Published:** 2013-12-18

**Authors:** H. Jang, Y. H. Kim, M. K. Kim, K. H. Lee, S. Jeon

**Affiliations:** ^1^Cutigen Research Institute, Tego Science Inc., Daerung Technotown III, 448 Gasan-Dong, Gumcheon-Gu, Seoul 153-772, Republic of Korea; ^2^Department of Biosystems & Biomaterials Science and Engineering, Seoul National University, Seoul 151-921, Republic of Korea

## Abstract

Lyophilized Cultured Epidermal Sheets (L-CES) have been reported to be as effective as the cryopreserved CES (F-CES) in treating skin ulcers. However, unlike F-CES, no preclinical study assessing wound-healing effects has been conducted for L-CES. The present study was set out to investigate the microstructure, cytokine profile, and wound-healing effects of L-CES in comparison to those of F-CES. Keratinocytes were cultured to prepare CES, followed by cryopreservation at −70°C and lyophilization. Under microscopic observation, intact cells with apparent intracellular junctions were observed in L-CES. The L-CES, like fresh CES, consisted of three to four well-maintained epidermal layers, as shown by the expression of keratins, involucrin, and p63. There were no differences in the epidermal layer or protein expression between L-CES and F-CES, and both CES were comparable to fresh CES. TGF-*α*, EGF, VEGF, IL-1*α*, and MMPs were detected in L-CES at levels similar to those in F-CES. In a mouse study, wounds treated with L-CES or F-CES completely healed at least 4 days faster than untreated wounds. CES-treated wounds completely healed by day 10, while the untreated wounds did not heal by day 14. Masson's trichrome staining showed that collagen deposition in the CES-treated wounds was highly increased in the dermis of the wound center compared to that in the control wounds. Thus, this study demonstrates that L-CES is as clinically effective as F-CES for wound treatment.

## 1. Introduction

CES have been used to treat cutaneous wounds such as burns and ulcers since the technologies to culture keratinocytes and to detach CES from the culture vessels were established [1–3].

It was shown in the late 1980s that CES of allogeneic keratinocytes, unlike their autologous counterpart, are not permanent, instead serving as a temporary biological dressing that releases proteins involved in the proliferation and migration of keratinocytes and fibroblasts. Proteins that act in concert to promote wound healing include growth factors such as bFGF, TGF-*α*, TGF-*β*, EGF, VEGF, and PDGF, cytokines such as IL-1*α*, IL-6, and IL-8, and extracellular matrix proteins such as collagen, laminin, tenascin, and fibronectin [4–6].

Allogeneic CES, prepared from a cell bank established from an infant skin biopsy, have advantages over autologous CES in that the time taken to obtain CES from primary culture is much shorter compared to autologous CES. In order for the allogeneic CES to be readily available, however, it is necessary to store them for some period of time without compromising their biological nature.

Studies have demonstrated that the F-CES are as effective as freshly prepared CES in treating partial-thickness wounds [7, 8]. Furthermore, L-CES were also shown to successfully treat chronic leg ulcers [9, 10]. Because lyophilization seems to provide a convenient and cost-effective way to prepare ready-made CES on a large scale, we were curious to know whether the wound-healing effects of L-CES are comparable to F-CES.

## 2. Materials and Methods

### 2.1. Preparation of F-CES and L-CES

Human keratinocytes were cultured as described in Barrandon and Green to form a sheet [11] that was detached from the culture flask using dispase [2]. For cryopreservation, the resulting CES were treated with a cryoprotectant containing human serum albumin in DMEM and then frozen at −70°C. Before lyophilization, CES were treated with a lyoprotectant of 5% polyethylene glycol (PEG), 1% trehalose, and cryoprotectant and then frozen at −40°C. More than 12 hours later, lyophilization was performed through a gradual increase in temperature at the rate of 1°C per minute from −40°C to 25°C under vacuum pressure of less than 10 mTorr in a lyophilizer (Ilshin Biobase, Seoul, South Korea).

### 2.2. Microstructural Observation of CES

The sheet surface of CES was observed using a field-emission scanning electron microscope (FE-SEM) (SUPRA 55VP, Carl Zeiss, Oberkochen, Germany). The cross sections of CES were examined for cell-cell junctions with a transmission electron microscope (TEM) (JEM1010, JEOL, Akishima, Tokyo, Japan).

### 2.3. Immunofluorescence Staining (IF)

Monoclonal antibodies raised against K1 (Abcam, Cambridge, MA, USA), K14 (AbD Serotec, Raleigh, NC, USA), involucrin (Sigma-Aldrich, St. Louis, MO, USA), and p63 (BD Pharmingen, San Jose, CA, USA) were used with secondary antibodies including anti-mouse IgG conjugated with either FITC (Jackson Immuno Research Laboratories Inc., West Grove, PA, USA) or rhodamine (Invitrogen, Grand Island, NY, USA). A mounting medium containing DAPI (Vector, Burlingame, CA, USA) was used for nuclear staining. IF images were observed with an Anaxioskop 40 FL Microscope (Carl Zeiss).

### 2.4. ELISA Assay

Protein extracts from CES were prepared by repeated freezing and thawing followed by homogenization. Commercially available ELISA kits were used according to the manufacturer's instructions (R&D Systems, Minneapolis, MN, USA) to measure the levels of proteins involved in wound healing.

### 2.5. *In Vivo* Wound-Healing Assay

All animal experiments were approved by the Seoul National University Institutional Care and Use Committee (approval number: SNU-120116-4). The wound-healing assay was performed using 8~12-week-old ICR mice [12]. Specifically, two full-thickness wounds 1.2 cm in diameter were made on the back of each mouse by excising the skin and were covered with either F-CES or L-CES. Vaseline gauze was applied in all groups, and the control wound was covered with Vaseline gauze only. The wound was treated with each graft once for 14 days without graft changes or additional treatment. The animals were sacrificed at days 4, 7, 10, and 14 after injury for histological analysis. Complete wounds were isolated, fixed overnight in 10% formalin, and embedded in paraffin. To compare collagen deposition, Masson's trichrome staining was performed on the paraffin sections.

### 2.6. Statistical Analysis

Student's *t*-test was used to compare the differences in protein levels between F-CES and L-CES and in wound-healing assay between F-CES or L-CES and control. *P* values <0.05 were considered significant. 

## 3. Results

### 3.1. Epidermal Structure of L-CES

**Figure 1:**
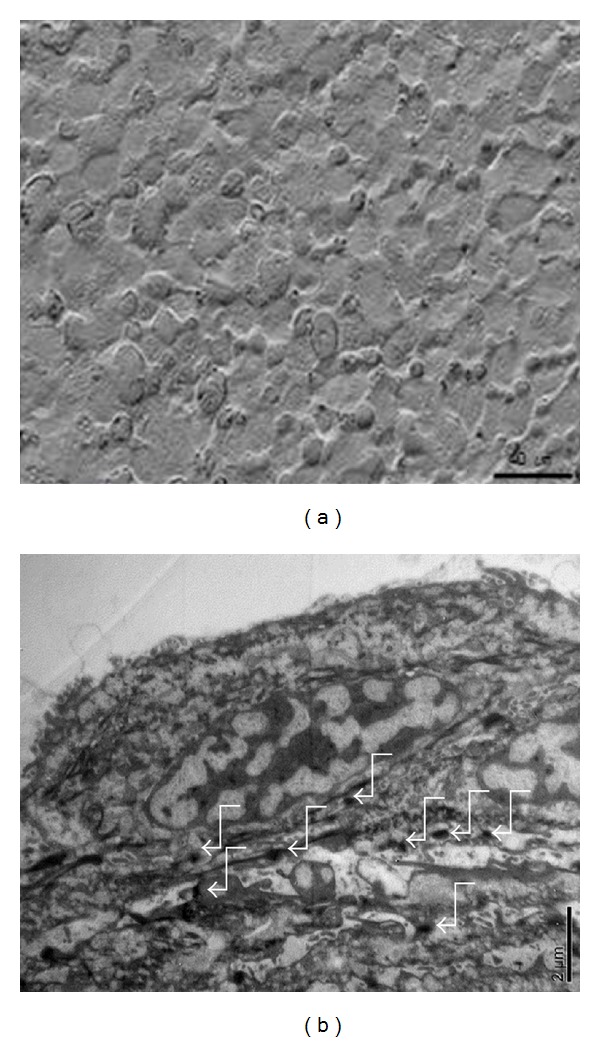
Microstructures of L-CES. (a) Top view of L-CES by the scanning electron microscope (SEM) and (b) cross-sectional view of L-CES by the transmission electron microscope (TEM). Desmosomes are indicated by white arrows. Scale bar: SEM 40 *μ*m, TEM 2 *μ*m.

**Figure 2:**
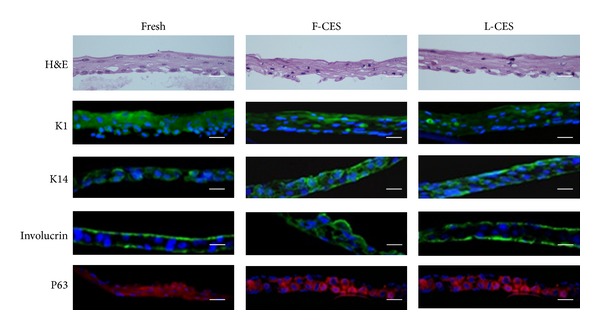
H&E and immunofluorescence staining of fresh CES, F-CES, and L-CES. K14: basal cell marker, K1 and involucrin: suprabasal cell marker, and p63: keratinocyte stem cell marker. Nuclei were stained blue with DAPI. Magnification: ×400 (H&E) and ×400 (IF staining). Scale bar: 25 *μ*m.

To prepare CES that are suitable for long-term storage and convenient handling, attempts to lyophilize CES were undertaken under the conditions described in Section 2. CES have been shown to be mainly composed of basal and spinous keratinocytes adhered via desmosomes and other adhesion proteins such as E-cadherin and *β*-catenin [13]. The microstructure of L-CES, as determined by scanning electron microscopy (SEM), appeared to be intact with apparent cell-cell junctions observed by TEM (Figure 1). The hematoxylin and eosin (H&E)-stained cross sections of L-CES revealed epidermal characteristics similar to both fresh CES and F-CES (Figure 2). In the epidermis, three to four cell layers were observed. The basal layers were positive for K14 and p63, and suprabasal layers were positive for K1 and involucrin by immunofluorescence staining. These data indicate that the lyophilization process did not harm the CES at least from a morphological point of view.

### 3.2. Growth Factors, Cytokines, and MMPs in L-CES

**Figure 3:**
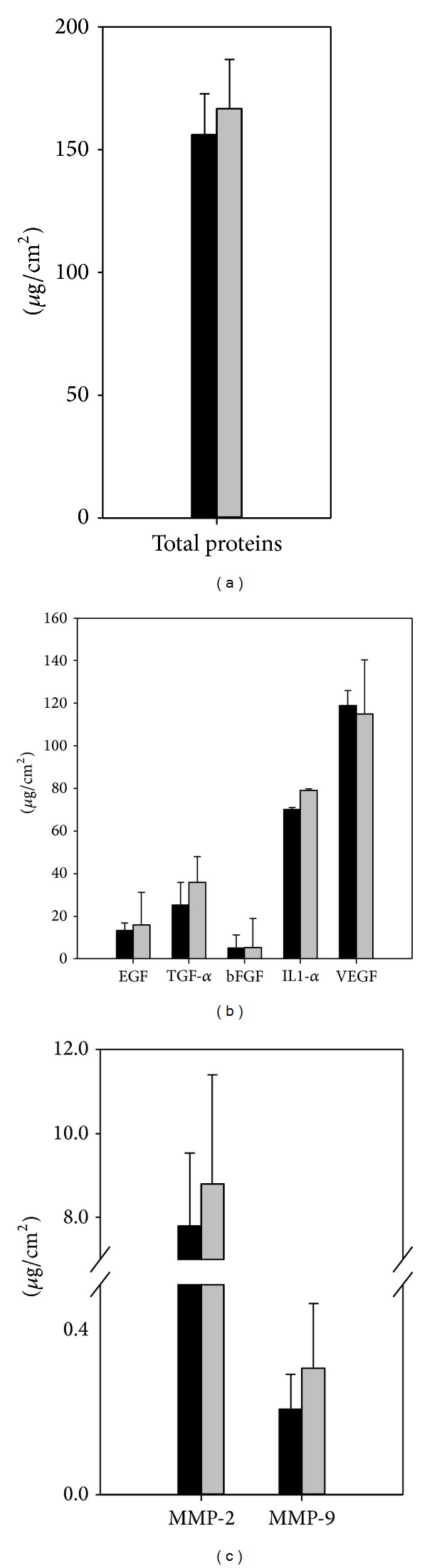
Levels of proteins measured from F-CES and L-CES. (a) Total proteins. (b) Growth factors and cytokines. (c) MMPs. The mean values for F-CES and L-CES, calculated from three independent experiments, are shown by black and grey bars, respectively. There was no statistical significance (*P* < 0.05).

Allogeneic CES have paracrine effects on wounds that are mediated by the release of proteins. The presence of the paracrine factors TGF-*α*, bFGF, EGF, VEGF, and IL-1*α* in L-CES was investigated. ELISA data showed that VEGF and IL-1*α* were detected at high levels in L-CES that were comparable to those in F-CES. TGF-*α*, EGF, and bFGF were also detected in both F-CES and L-CES (Figure 3). Therefore, CES, regardless of their physical states (frozen or lyophilized), are expected to exert similar paracrine effects on wound healing. In addition, matrix metalloproteases (MMPs) such as MMP-2 and MMP-9 were found in L-CES at similar or higher levels than in F-CES, although the difference was not statistically significant (Figure 3). These enzymes are known to play an important role in controlling the overproduction of collagen and thus in reducing hypertrophic scars *in vivo *[14]. These results lead us to believe that lyophilization does not compromise the protein levels of CES involved in wound healing and scar formation.

### 3.3. *In Vivo* Wound Healing in Mice

**Figure 4:**
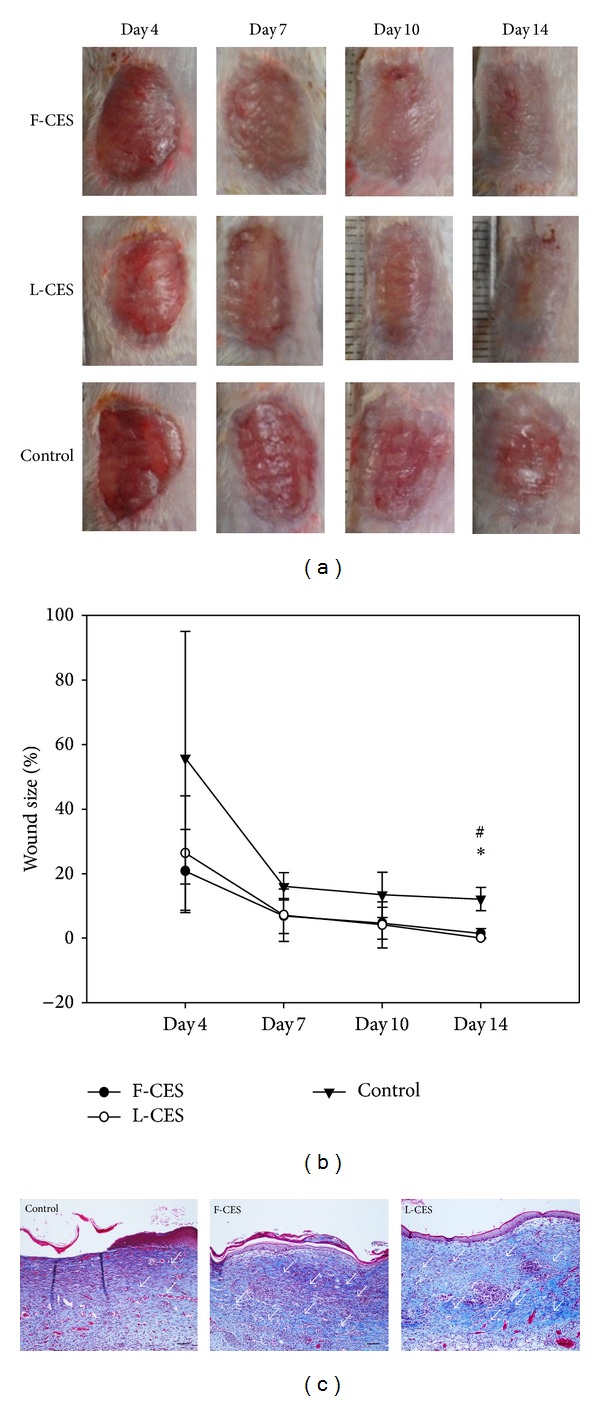
Effects of L-CES on *in vivo* wound healing in mice compared to F-CES. (a) Wounds observed on days 4, 7, 10, and 14 after wounding. (b) A graph showing the percentage of unhealed area over initial wound size as a function of time for treatment. Statistical significances between F-CES (*P < 0.05, *P* = 0.04) or L-CES (^#^ *P* < 0.05, *P* = 0.03) and control were indicated. (c) Masson's trichrome staining of cross-sectioned biopsies taken from wounds on day 14. Collagen deposition was shown by white arrows. Magnification: ×100, scale bar: 100 *μ*m.

To compare the wound-healing effect of L-CES and F-CES, two full-thickness wounds were created on the back of ICR mice and were either treated with F-CES or L-CES or were left untreated (Figure 4). We measured nonreepithelialized area, and the size of the unhealed fraction of the wound was measured at 4, 7, 10, and 14 days after wounding. The percentage of unhealed area over the initial wound size was calculated to evaluate the healing effect. Wound size was effectively decreased in wounds treated with either F-CES or L-CES, to 20.8% and 26.4%, respectively, whereas it was 55.9% in untreated controls on day 4 (Figures 4(a) and 4(b)).

By day 7, the wounds treated with either form of CES were reduced to less than 10% and were half the size of the control wounds. Even on the 10th day, unhealed area for wounds treated with either CES was 1.4%, which can be regarded as complete closure (<2.0%) in our experimental setting. A continued decrease in size to complete closure was observed by day 14 (0.1%). In contrast, the control wounds were not fully closed, with a 12.1% unhealed fraction on day 14. Therefore, healing capacity of F-CES (*P* = 0.04) and L-CES (*P* = 0.03) was significant compared to that of untreated control on day 14. Reepithelialization was shown in the wound center in both F-CES- and L-CES-treated wounds at day 14, whereas it was not observed in the wound center in untreated controls (Figure 4(c)). Finally, CES-treated wounds completely healed by day 10, while the untreated wounds had only partially healed by day 14.

Collagen deposition was detected at high levels in both F-CES- and L-CES-treated wounds. Collagen deposition in the wound center was significantly increased in both F-CES- and L-CES- treated wounds compared to untreated controls. It should be emphasized that we did not notice any difference between L-CES and F-CES in their ability to promote wound healing in mice.

## 4. Discussion 

Since the technologies to culture keratinocytes *in vitro *to form a sheet and to detach it from culture flasks were established in late 1970s, CES have been used to treat severely burned patients by providing keratinocyte stem cells for reepithelialization of the wounded areas [3, 15]. CES composed of allogeneic keratinocytes, in particular, could be defined as a biological dressing that exerts its wound-healing effects by releasing multiple proteins including growth factors, cytokines, and extracellular matrix proteins. When epidermal and/or follicular stem cells remain in or around a wound, CES have been reported to stimulate these stem cells to proliferate, migrate, and eventually cover the entire wound [4–6]. It was evident that the regenerated epithelium consisted of the patient's own keratinocytes instead of those provided by the CES [16, 17].

In order for CES to be readily available, different methods of cryopreserving them for a substantial period of time without compromising their biological activities have been developed. The resulting cryopreserved CES have been clinically shown to have healing effects similar to freshly prepared CES [18] and have been successfully commercialized (Kaloderm, Tego Science Inc., Seoul, South Korea). Although cryopreservation can make it possible to culture CES in quantity and to apply them immediately to the wounds whenever needed, it is cumbersome to maintain deep freezers equipped with electrical generator systems in case of power failure and to deliver F-CES over distances using dry ice. Once these disadvantages are overcome, the market potential of CES is expected to rise tremendously due to their outstanding efficacy. A few studies have described the clinical application of L-CES to skin ulcers as an alternative to F-CES [9, 10]. However, those studies are limited in that L-CES have never been characterized *in vitro* in terms of their structural integrity or biological characteristics. In our study, the structural and morphological integrity of L-CES were examined. The cross sections of L-CES were comparable to those of both fresh CES and F-CES in terms of their expression of epidermal marker proteins such as K14, K1, and involucrin. In addition, the levels of proteins, including growth factors and matrix metalloproteases, known to be involved in wound healing were similar in L-CES and F-CES. These results indicate that the lyophilization process did not compromise the biological activity of the CES. These *in vitro* results were further confirmed by an *in vivo* wound-healing study. In mice, both F-CES and L-CES demonstrated similar healing effects on full-thickness wounds. Wound-healing process involves reepithelialization, contraction, and connective tissue deposition, and these steps of wound healing act concurrently. We demonstrated that reepithelialization in histological analysis was completed in wounds treated with L-CES or F-CES while it was not completed in untreated wound at day 14. Wounds treated with L-CES or F-CES revealed that the collagen deposition was abundant in Masson's trichrome staining. Therefore, both *in vitro* and *in vivo* data suggest that lyophilization, similar to cryopreservation, is a useful option to preserve the wound-healing proteins and, therefore, the healing effect of CES. In addition, L-CES are comparable to lyophilized keratinocyte lysates in that the same set of proteins is implicated and clinically active in wound healing [19, 20]. However, L-CES are presumed to be more stable because the integrity of cells is maintained after lyophilization, although cells could be partially lysed.

Lyophilization is one of the most practical methods of long-term preservation at room temperature in a variety of industries including food, agriculture, cosmetics, and medicine. However, the lyophilization of biological products has been confined to purified materials, such as growth factors and hormones, the microorganisms used for forage and vaccines, because they are likely to be functionally unaffected by the process in comparison to mammalian cells [21]. Trial and error was used to establish optimal conditions under which CES without changing their biological activities in this study were lyophilized (publication in preparation). In brief, we treated CES with an undisclosed combination of polyethylene glycol (PEG), proteins, and sugars and lyophilized them under the conditions described in Section 2. These lyophilization conditions allowed CES to be biologically stable over a period of months at room temperature. Further studies remain to be conducted to fine-tune the lyophilization conditions and prolong the allowable storage of L-CES at room temperature.

## 5. Conclusion

L-CES have been reported to be as clinically effective as F-CES in wound healing. In the present study, L-CES were analyzed to show that their structural integrity, epidermal structure, cytokine contents, and wound-healing capacity are unchanged by the lyophilization process. Therefore, we conclude that lyophilization may be a means to have CES readily available at room temperature without affecting their biological activities in wound healing.
